# A Parallel Decoding Algorithm for Short Polar Codes Based on Error Checking and Correcting

**DOI:** 10.1155/2014/895782

**Published:** 2014-07-23

**Authors:** Yingxian Zhang, Xiaofei Pan, Kegang Pan, Zhan Ye, Chao Gong

**Affiliations:** Laboratory of Satellite Communications, College of Communications Engineering, PLA University of Science and Technology, Nanjing 210007, China

## Abstract

We propose a parallel decoding algorithm based on error checking and correcting to improve the performance of the short polar codes. In order to enhance the error-correcting capacity of the decoding algorithm, we first derive the* error-checking equations* generated on the basis of the frozen nodes, and then we introduce the method to check the errors in the input nodes of the decoder by the solutions of these equations. In order to further correct those checked errors, we adopt the method of modifying the probability messages of the error nodes with constant values according to the maximization principle. Due to the existence of multiple solutions of the* error-checking equations*, we formulate a CRC-aided optimization problem of finding the optimal solution with three different target functions, so as to improve the accuracy of error checking. Besides, in order to increase the throughput of decoding, we use a parallel method based on the decoding tree to calculate probability messages of all the nodes in the decoder. Numerical results show that the proposed decoding algorithm achieves better performance than that of some existing decoding algorithms with the same code length.

## 1. Introduction

Due to the ability of achieving Shannon capacity and its low encoding and decoding complexity, the polar codes have received much attention in recent years [1–20]. However, compared to some original coding schemes such as LDPC and Turbo codes, the polar codes have a remarkable drawback; that is, the performance of the codes in the finite length regime is limited [2, 3]. Hence, researchers have proposed many decoding algorithms to improve the performance of the codes [4–19].

In [4, 5], a list successive-cancelation (SCL) decoding algorithm was proposed with consideration of *L* successive-cancelation (SC) [1] decoding paths, and the results showed that performance of SCL was very close to that of maximum-likelihood (ML) decoding. Then, in [6], another decoding algorithm derived from SC called stack successive-cancelation (SCS) was introduced to decrease the time complexity of the SCL. In particular, with CRC aided, SCL yielded better performance than that of some Turbo codes, as shown in [7]. However, due to the serial processing nature of the SC, the algorithms in [4–7] suffered a low decoding throughput and high latency. Based on this observation, some improved versions of SC were proposed with the explicit aim to increase throughput and reduce the latency without sacrificing error-rate performance, such as simplified successive-cancellation (SSC) [8], maximum-likelihood SSC (ML-SSC) [9], and repetition single parity check ML-SSC (RSM-SSC) [10, 11]. Besides those SC based algorithms, researchers had also investigated some other algorithms. In [12, 13], the ML and maximum a posteriori (MAP) decoding were proposed for the short polar codes. And in [14], a linear programming decoder was introduced for the binary erase channels (BECs). With the factor graph representation of polar codes [15], authors in [16, 17] showed that belief propagation (BP) polar decoding had particular advantages with respect to the decoding throughput, while the performance was better than that of the SC and some improved SC decoding. What is more is that, with the minimal stopping set optimized, results of [18, 19] had shown that the error floor performance of polar codes was superior to that of LDPC codes.

Indeed, all the decoding algorithms in [4–19] can improve the performance of polar codes to a certain degree. However, as the capacity achieving coding scheme, the results of those algorithms are disappointing. Hence, we cannot help wondering why the performance of the polar codes with finite length is inferior to that of the existing coding schemes and how we can improve it. To answer the questions, we need to make a further analysis of those decoding algorithms in [4–19].

For the decoding algorithms with serial processing, there has been the problem of error propagation except the low decoding throughput and high latency [20, 21]. That is to say, errors which occurred in the previous node will lead to the error decoding of the later node. However, none of the existing serial processing algorithms has considered this observation. Furthermore, it is noticed from the factor graph of polar codes in [15] that the degree of the check or variable nodes in the decoder is 2 or 3, which will weaken the error-correcting capacity of the decoding, as compared to the LDPC codes with the average degree usually greater than 3 [22, 23]. Hence, the performance of the polar codes is inferior to that of LDPC codes with the same length [18, 19]. What is more is that BP polar decoding needs more iterations than that of LDPC codes, as shown in [16, 17, 22, 23]. Therefore, in order to improve the performance of a decoding algorithm for polar codes, it is important to enhance the error-correcting capacity of the algorithm.

Motivated by aforementioned observations, we propose a parallel decoding algorithm for short polar codes based on error checking and correcting in this paper. We first classify the nodes of the proposed decoder into two categories:* information nodes* and* frozen nodes*, values of which are determined and independent of decoding algorithms. Then, we introduce the method to check the errors in the input nodes of the decoder, with the solutions of the* error-checking equations* generated based on the frozen nodes. To correct those checked errors, we modify the probability messages of the error nodes with constant values according to the maximization principle. Through delving the error-checking equations solving problem, we find that there exist multiple solutions for those equations. Hence, as to check the errors as accurately as possible, we further formulate a CRC-aided optimization problem of finding the optimal solution of the error-checking equations with three different target functions. Besides, we also use a parallel method based on the* decoding tree representations of the nodes* to calculate probability messages in order to increase the throughput of decoding. The main contributions of this paper can be summarized as follows.

- An error-checking algorithm for polar decoding based on the error-checking equations solving is introduced; furthermore, as to enhance the accuracy of the error checking, a CRC-aided optimization problem of finding the optimal solution is formulated.
- To correct the checked errors, we propose a method of modifying the probability messages of the error nodes according to the maximization principle.
- In order to improve the throughput of the decoding, we propose a parallel probability messages calculating method based on the decoding tree representation of the nodes.
- The whole procedure of the proposed decoding algorithm is described with the form of pseudocode, and the complexity of the algorithm is also analyzed.

The finding of this paper suggests that, with the error checking and correcting, the error-correcting capacity of the decoding algorithm can be enhanced, which will yield a better performance at cost of certain complexity. Specifically, with the parallel probability messages calculating, the throughput of decoding is higher than the serial process based decoding algorithms. All of these results are finally proved by our simulation work.

The remainder of this paper is organized as follows. In Section 2, we explain some notations and introduce certain preliminary concepts used in the subsequent sections. And in Section 3, the method of the error checking for decoding based on the error-checking equations is described in detail. In Section 4, we introduce the methods of probability messages calculating and error correcting, and after the formulation of the CRC-aided optimization problem of finding the optimal solution, the proposed decoding algorithm with the form of pseudocode is presented. Then, the complexity of our algorithm is analyzed.  Section 5 provides the simulation results for the complexity and bit error performance. Finally, we make some conclusions in Section 6.

## 2. Preliminary

### 2.1. Notations

In this work, the blackboard bold letters, such as *X*, denote the sets, and |*X*| denotes the number of elements in *X*. The notation *u*_0_^*N*−1^ denotes an *N*-dimensional vector (*u*_0_, *u*_1_,…, *u*_*N*−1_), and *u*_*i*_^*j*^ indicates a subvector (*u*_*i*_, *u*_*i*+1_,…, *u*_*j*−1_, *u*_*j*_) of *u*_0_^*N*−1^, 0 ≤ *i*, *j* ≤ *N* − 1. When *i* > *j*, *u*_*i*_^*j*^ is an empty vector. Further, given a vector set *U*, vector ${\vec{u}}_{i}$ is the *i*th element of *U*.

The matrixes in this work are denoted by bold letters. The subscript of a matrix indicates its size; for example, **A**_*N*×*M*_ represents an *N* × *M* matrix **A**. Specifically, the square matrixes are written as **A**_*N*_, size of which is *N* × *N*, and **A**_*N*_^−1^ is the inverse of **A**_*N*_. Furthermore, the Kronecker product of two matrixes **A** and **B** is written as **A** ⊗ **B**, and the *n*th Kronecker power of **A** is **A** ^⊗*n*^.

During the procedure of the encoding and decoding, we denote the intermediate node as *v*(*i*, *j*), 0 ≤ *i* ≤ *n*, 0 ≤ *j* ≤ *N* − 1, where *N* = 2^*n*^ is the code length. Besides, we also indicate the probability values of the intermediate node *v*(*i*, *j*) being equal to 0 or 1 as *p*_*v*(*i*,*j*)_(0) or *p*_*v*(*i*,*j*)_(1).

Throughout this Paper, “⊕” denotes the Modulo-Two Sum, and “∑_*i*=0_^*M*^ ⊕ *x*_*i*_” means “*x*_0_ ⊕ *x*_1_ ⊕ ,…, ⊕*x*_*M*_”.

### 2.2. Polar Encoding and Decoding

A polar coding scheme can be uniquely defined by three parameters: block-length *N* = 2^*n*^, code rate *R* = *K*/*N*, and an information set *I* ⊂ *N* = {0,1,…, *N* − 1}, where *K* = |*I*|. With these three parameters, a source binary vector *u*_0_^*N*−1^ consisting of *K* information bits and *N* − *K* frozen bits can be mapped a codeword *x*_0_^*N*−1^ by a linear matrix **G**_*N*_ = **B**_*N*_ **F**_2_^⊗*n*^, where $F_{2}=\left[\begin{array}{cc} 1 & 0\\ 1 & 1 \end{array}\right]$, **B**_*N*_ is a bit-reversal permutation matrix defined in [1], and *x*_0_^*N*−1^ = *u*_0_^*N*−1^ **G**_*N*_.

**Figure 1:**
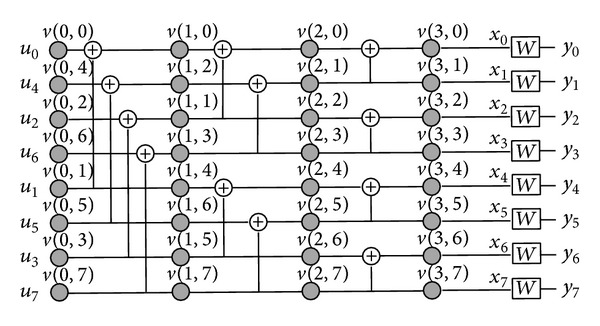
Construction of the polar encoding with length *N* = 8.

In practice, the polar encoding can be completed with the construction shown in Figure 1, where the gray circle nodes are the intermediate nodes. And the nodes in the leftmost column are the input nodes of encoder, values of which are equal to binary source vector; that is, *v*(0, *i*) = *u*_*i*_, while the nodes in the rightmost column are the output nodes of encoder, *v*(*n*, *i*) = *x*_*i*_. Based on the construction, a codeword *x*_0_^7^ is generated by the recursively linear transformation of the nodes between adjacent columns.

After the procedure of the polar encoding, all the bits in the codeword *x*_0_^*N*−1^ are passed to the *N*-channels, which are consisted of *N* independent channels of *W*, with a transition probability of *W*(*y*_*i*_∣*x*_*i*_), where *y*_*i*_ is *i*th element of the received vector *y*_0_^*N*−1^.

At the receiver, the decoder can output the estimated codeword ${\hat{x}}_{0}^{N-1}$ and the estimated source binary vector ${\hat{u}}_{0}^{N-1}$ with different decoding algorithms [1–19]. It is noticed from [1–19] that the construction of all the decoders is the same as that of the encoder; here, we make a strict proof for that with the mathematical formula in the following theorem.

**Theorem 1 .**

For the generation matrix of the a polar code **G**_*N*_, there exists

(1) $$\begin{array}{c} G_{N}^{-1}=G_{N}. \end{array}$$

That is to say, for the decoding of the polar codes, one will have

(2) $$\begin{array}{c} {\hat{u}}_{0}^{N-1}={\hat{x}}_{0}^{N-1}G_{N}^{-1}={\hat{x}}_{0}^{N-1}G_{N}, \end{array}$$

where **G**_*N*_^−1^ is construction matrix of the decoder.

**Proof**

The proof of Theorem 1 is based on the matrix transformation, which is shown detailedly in Appendix A.

Hence, as for the polar encoder shown in Figure 1, there is

(3) $$\begin{array}{c} G_{8}=G_{8}^{-1}=\left[\begin{array}{cccccccc} 1 & 0 & 0 & 0 & 0 & 0 & 0 & 0\\ 1 & 0 & 0 & 0 & 1 & 0 & 0 & 0\\ 1 & 0 & 1 & 0 & 0 & 0 & 0 & 0\\ 1 & 0 & 1 & 0 & 1 & 0 & 1 & 0\\ 1 & 1 & 0 & 0 & 0 & 0 & 0 & 0\\ 1 & 1 & 0 & 0 & 1 & 1 & 0 & 0\\ 1 & 1 & 1 & 1 & 0 & 0 & 0 & 0\\ 1 & 1 & 1 & 1 & 1 & 1 & 1 & 1 \end{array}\right]. \end{array}$$

**Figure 2:**
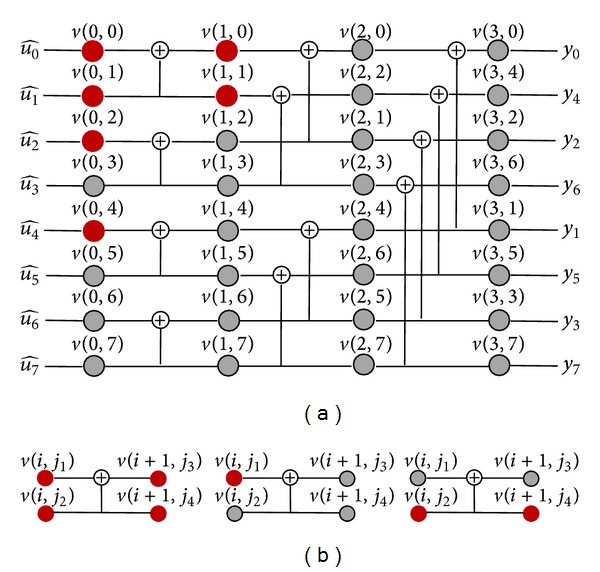
(a) Construction of polar decoding with code length *N* = 8. (b) Basic process units of the polar decoder.

Furthermore, we have the construction of the decoder as shown in Figure 2(a), where nodes in the rightmost column are the input nodes of the decoder, and the output nodes are the nodes in the leftmost column. During the procedure of the decoding, the probability messages of the received vector are recursively propagated from the rightmost column nodes to the leftmost column nodes. Then, the estimated source binary vector ${\hat{u}}_{0}^{7}$ can be decided by

(4) $$\begin{array}{c} {\hat{u}}_{i}=\{\begin{array}{cc} 0, & p_{v(0,i)}\left(0\right)>p_{v(0,i)}\left(1\right)\\ 1, & \text{otherwise}. \end{array} \end{array}$$

In fact, the input probability messages of the decoder depend on the transition probability *W*(*y*_*i*_∣*x*_*i*_) and the received vector *y*_0_^7^; hence, there is

(5) pv(n,i)(0)=W(yi ∣ xi=0),pv(n,i)(1)=W(yi ∣ xi=1).

For convenience of expression, we will write the input probability messages *W*(*y*_*i*_∣*x*_*i*_ = 0) and *W*(*y*_*i*_∣*x*_*i*_ = 1) as *q*_*i*_(0) and *q*_*i*_(1), respectively, in the rest of this paper. Therefore, we further have

(6) pv(n,i)(0)=qi(0),pv(n,i)(1)=qi(1).

### 2.3. Frozen and Information Nodes

In practice, due to the input of frozen bits [1], values of some nodes in the decoder are determined, which are independent of the decoding algorithm, as the red circle nodes illustrated in Figure 2(a) (code construction method is the same as [1]). Based on this observation, we classify the nodes in the decoder into two categories: the nodes with determined values are called* frozen nodes*, and the other nodes are called* information nodes*, as the gray circle nodes shown in Figure 2(a). In addition, with the basic process units of the polar decoder shown in Figure 2(b), we have the following lemma.

**Lemma 2 .**

For the decoder of a polar code with rate *R* < 1, there must exist some frozen nodes, the number of which depends on the information set *I*.

**Proof**

The proof of Lemma 2 can be easily finished based on the process units of the polar decoder as shown in Figure 2(b), where *v*(*i*, *j*_1_), *v*(*i*, *j*_2_), *v*(*i* + 1, *j*_3_), and *v*(*i* + 1, *j*_4_) are the four nodes of the decoder.

Lemma 2 has shown that, for a polar code with rate *R* < 1, the frozen nodes are always existing; for example, the frozen nodes in Figure 2(a) are *v*(0,0), *v*(1,0), *v*(0,1), *v*(1,1), *v*(0,2), and *v*(0,4). For convenience, we denote the frozen node set of a polar code as *V*_*F*_, and we assume that the default value of each frozen node is 0 in the subsequent sections.

### 2.4. Decoding Tree Representation

**Figure 3:**
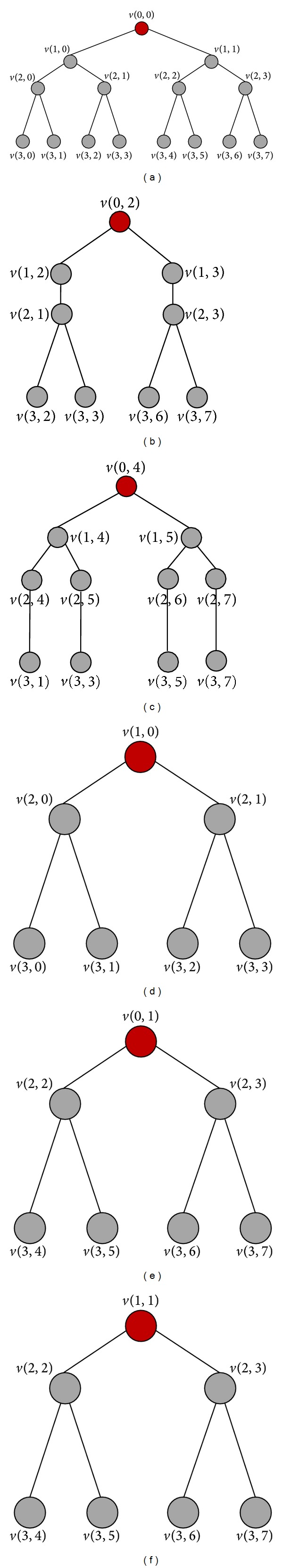
The decoding trees for the nodes *v*(0,0), *v*(0,1), *v*(1,0), *v*(1,1), *v*(0,2), and *v*(0,4).

It can be found from the construction of the decoder in Figure 2(a) that the decoding of a node *v*(*i*, *j*) can be equivalently represented as a binary decoding tree with some input nodes, where *v*(*i*, *j*) is the root node of that tree, and the input nodes are the leaf nodes. The height of a decoding tree is as most as log_2_ *N*, and each of the intermediate node has one or two son nodes. As illustrated in Figure 3, the decoding trees for frozen nodes *v*(0,0), *v*(0,1), *v*(0,2), *v*(0,4), *v*(1,0), and *v*(1,1) in Figure 2(a) are given.

During the decoding procedure, probability messages of *v*(0,0), *v*(0,1), *v*(0,2), *v*(0,4), *v*(1,0), and *v*(1,1) will strictly depend on the probability messages of the leaf nodes as the bottom nodes shown in Figure 3. In addition, based on the (2), we further have

(7) v(0,0)=∑i=07⊕v(3,i)v(1,0)=v(3,0)⊕v(3,1)⊕v(3,2)⊕v(3,3)v(0,1)=v(3,4)⊕v(3,5)⊕v(3,6)⊕v(3,7)v(1,1)=v(3,4)⊕v(3,5)⊕v(3,6)⊕v(3,7)v(0,2)=v(3,2)⊕v(3,3)⊕v(3,6)⊕v(3,7)v(0,4)=v(3,1)⊕v(3,3)⊕v(3,5)⊕v(3,7).

To generalize the decoding tree representation for the decoding, we introduce the following Lemma.

**Lemma 3 .**

In the decoder of a polar code with length *N* = 2^*n*^, there is a unique decoding tree for each node *v*(*i*, *j*), the leaf nodes set of which is indicated as *V*_*v*(*i*,*j*)_^*L*^. And if *j* ≠ *N* − 1, the number of the leaf nodes is even; that is,

(8)       v(i,j)=∑k=0M/2⊕v(n,j2k),0≤j2k≤N−1,  v(n,j2k)∈Vv(i,j)L,

where *M* = |*V*_*v*(*i*,*j*)_^*L*^| and (*M*mod⁡2) = 0. While if *j* = *N* − 1, *M* is equal to 1, and it is true that

(9) $$\begin{array}{c} v\left(i,N-1\right)=v\left(n,N-1\right). \end{array}$$

**Proof**

The proof of Lemma 3 is based on (2) and construction of the generation matrix. It is easily proved that, except the last column (only one “1” element), there is an even number of “1” elements in all the other columns of **F**_2_^⊗*n*^. As **B**_*N*_ is a bit-reversal permutation matrix, which is generated by permutation of rows in **I**_*N*_, hence, the generation matrix **G**_*N*_ has the same characteristic as **F**_2_^⊗*n*^ (see the proof of Theorem 1). Therefore, (8) and (9) can be easily proved by (2).

Lemma 3 has clearly shown the relationship between the input nodes and other intermediate nodes of the decoder, which is useful for error checking and probability messages calculation introduced in the subsequent sections.

## 3. Error Checking for Decoding

As analyzed in Section 1, the key problem to improve the performance of polar codes is to enhance the error-correcting capacity of the decoding. In this section, we will show how to achieve the goal.

### 3.1. Error Checking by the Frozen Nodes

It is noticed from Section 2.3 that the values of the frozen nodes are determined. Hence, if the decoding is correct, the probability messages of any frozen node *v*(*i*, *j*) must satisfy the condition of *p*_*v*(*i*,*j*)_(0) > *p*_*v*(*i*,*j*)_(1) (the default value of frozen nodes is 0), which is called* reliability condition* throughout this paper. While in practice, due to the noise of received signal, there may exist some frozen nodes unsatisfying the reliability condition, which indicates that there must exist errors in the input nodes of the decoder. Hence, it is exactly based on this observation that we can check the errors during the decoding. To further describe detailedly, a theorem is introduced to show the relationship between the reliability condition of the frozen nodes and the errors in the input nodes of the decoder.

**Theorem 4 .**

For any frozen node *v*(*i*, *j*) with a leaf node set *V*_*v*(*i*,*j*)_^*L*^, if the probability messages of *v*(*i*, *j*) do not satisfy the reliability condition during the decoding procedure, there must exist an odd number of error nodes in *V*_*v*(*i*,*j*)_^*L*^; otherwise, the error number will be even (including 0).

**Proof**

For the proof of Theorem 4 see Appendix B for detail.

Theorem 4 has provided us an effective method to detect the errors in the leaf nodes set of the frozen node. For example, if the probability messages of the frozen node *v*(0,0) in Figure 2 do not satisfy the reliability condition, that is, *p*_*v*(0,0)_(0) ≤ *p*_*v*(0,0)_(1), it can be confirmed that there must exist errors in the set of {*v*(3,0), *v*(3,1),…, *v*(3,7)}, and the number of these errors may be 1 or 3 or 5 or 7. That is to say, through checking the reliability condition of the frozen nodes, we can confirm existence of the errors in the input nodes of the decoder, which is further presented as a corollary.

**Corollary 5 .**

For a polar code with the frozen node set *V*_*F*_, if ∃*v*(*i*, *j*) ∈ *V*_*F*_ and *v*(*i*, *j*) does not satisfy the reliability condition, there must exist errors in the input nodes of decoder.

**Proof**

The proof of Corollary 5 is easily completed based on Theorem 4.

Corollary 5 has clearly shown that, through checking the probability messages of each frozen node, errors in the input nodes of decoder can be detected.

### 3.2. Error-Checking Equations

As aforementioned, errors in the input nodes can be found with probability messages of the frozen node, but there still is a problem, which is how to locate the exact position of each error. To solve the problem, a parameter called* error indicator* is defined for each input node of the decoder. And for the input node *v*(*n*, *i*), the error indicator is denoted as *c*_*i*_, value of which is given by

(10) $$\begin{array}{c} c_{i}=\{\begin{array}{cc} 1, & v\left(n,i\right)\;\text{is}\;\text{error}\\ 0, & \text{otherwise}. \end{array} \end{array}$$

That is to say, by the parameter of error indicator, we can determine whether an input node is error or not. Hence, the above problem can be transformed into how to obtain the error indicator of each input node. Motivated by this observation, we introduce another corollary of Theorem 4.

**Corollary 6 .**

For any frozen node *v*(*i*, *j*) with a leaf node set *V*_*v*(*i*,*j*)_^*L*^, there is

(11) (∑k=0M−1cik)mod⁡2=1, pv(i,j)(0)≤pv(i,j)(1)(∑k=0M−1cik)mod⁡2=0, otherwise,

where *M* = |*V*_*v*(*i*,*j*)_^*L*^|, *v*(*n*, *i*_*k*_) ∈ *V*_*v*(*i*,*j*)_^*L*^, and *N* = 2^*n*^ is code length. Furthermore, under the field of *GF*(2), (11) can be written as

(12) ∑k=0M−1⊕cik=1, pv(i,j)(0)≤pv(i,j)(1)∑k=0M−1⊕cik=0, otherwise.

**Proof**

The proof of Corollary 6 is based on Lemma 3 and Theorem 4, and here we ignore the detailed derivation process.

Corollary 6 has shown that the problem of obtaining the error indicator can be transformed to find solutions of (12) under the condition of (11). In order to introduce more specifically, here, we will take an example based on the decoder in Figure 2(a).

**Example 7 .**

We assume that frozen nodes *v*(0,0), *v*(1,0), *v*(0,2), and *v*(0,4) do not satisfy the reliability condition; hence, based on Theorem 4 and Corollary 6, there are equations as

(13) ∑i=07⊕ci=1‍c0⊕c1⊕c2⊕c3=1c4⊕c5⊕c6⊕c7=0c4⊕c5⊕c6⊕c7=0c2⊕c3⊕c6⊕c7=1c1⊕c3⊕c5⊕c7=1.

Furthermore, (13) can be written as matrix form, which is

(14) $$\begin{array}{c} E_{68}{\left(c_{0}^{7}\right)}^{T}=\left[\begin{array}{cccccccc} 1 & 1 & 1 & 1 & 1 & 1 & 1 & 1\\ 1 & 1 & 1 & 1 & 0 & 0 & 0 & 0\\ 0 & 0 & 0 & 0 & 1 & 1 & 1 & 1\\ 0 & 0 & 0 & 0 & 1 & 1 & 1 & 1\\ 0 & 0 & 1 & 1 & 0 & 0 & 1 & 1\\ 0 & 1 & 0 & 1 & 0 & 1 & 0 & 1 \end{array}\right]\left[\begin{array}{c} c_{0}\\ c_{1}\\ c_{2}\\ c_{3}\\ c_{4}\\ c_{5}\\ c_{6}\\ c_{7} \end{array}\right]={\left(\gamma_{0}^{5}\right)}^{T}, \end{array}$$

where $\gamma_{0}^{5}=\left(\begin{array}{cccccc} 1, & 1, & 0, & 0, & 1, & 1, \end{array}\right)$ and **E**_68_ is the coefficient matrix with size of 6 × 8. Therefore, by solving (14), we will get the error indicator vector of input nodes in Figure 2. In order to further generalize the above example, we provide a lemma.

**Lemma 8 .**

For a polar code with the code length *N*, code rate *R* = *K*/*N*, and frozen node set *V*_*F*_, we have the* error-checking equations* as

(15) $$\begin{array}{c} E_{MN}{\left(c_{0}^{N-1}\right)}^{T}={\left(\gamma_{0}^{M-1}\right)}^{T}, \end{array}$$

where *c*_0_^*N*−1^ is the error indicator vector and *M* = |*V*_*F*_|, *M* ≥ *N* − *K*. **E**_*MN*_ is called* error-checking matrix*, elements of which are determined by the code construction method, and *γ*_0_^*M*−1^ is called* error-checking vector*, elements of which depend on the probability messages of the frozen nodes in *V*_*F*_; that is, ∀*v*_*i*_ ∈ *V*_*F*_, 0 ≤ *i* ≤ *M* − 1, there is a unique *γ*_*i*_ ∈ *γ*_0_^*M*−1^ such that

(16) $$\begin{array}{c} \gamma_{i}=\{\begin{array}{cc} 1, & p_{v_{i}}\left(0\right)\leq p_{v_{i}}\left(1\right)\\ 0, & p_{v_{i}}\left(0\right)>p_{v_{i}}\left(1\right). \end{array} \end{array}$$

**Proof**

The proof of the Lemma 8 is based on (10)–(14), Lemma 3, and Theorem 4, which will be ignored here.

### 3.3. Solutions of Error-Checking Equations

Lemma 8 provides a general method to determine the position of errors in the input nodes by the error-checking equations. It is still needed to investigate the existence of solutions of the error-checking equations.

**Theorem 9 .**

For a polar code with code length *N* and code rate *R* = *K*/*N*, there is

(17) rank⁡(EMN)=rank⁡([EMN(γ0M−1)T])=N−K,

where $\left[\begin{array}{cc} E_{MN} & {\left(\gamma_{0}^{M-1}\right)}^{T} \end{array}\right]$ is the augmented matrix of (15) and rank⁡(**X**) is the rank of matrix **X**.

**Proof**

For the proof of Theorem 9 see Appendix C for detail.

It is noticed from Theorem 9 that there must exist multiple solutions for error-checking equations; therefore, we further investigate the general expression of solutions of the error-checking equations as shown in the following corollary.

**Corollary 10 .**

For a polar code with code length *N* and code rate *R* = *K*/*N*, there exists a transformation matrix **P**_*NM*_ in the field of *GF*(2) such that

(18) [EMN(γ0M−1)T] →PNM[IHAHK(γ¯0H−1)T0(M−H)H0(M−H)K0(M−H)×1],

where *H* = *N* − *K*, **A**_*HK*_ is the submatrix of transformation result of **E**_*MN*_, and ${\bar{\gamma }}_{0}^{H-1}$ is the subvector of transformation result of *γ*_0_^*M*−1^. Based on (18), the general solutions of error-checking equations can be obtained by

(19) $$\begin{array}{c} {\left({\hat{c}}_{0}^{N-1}\right)}^{T}=\left[\begin{array}{c} {\left({\hat{c}}_{K}^{N-1}\right)}^{T}\\ {\left({\hat{c}}_{0}^{K-1}\right)}^{T} \end{array}\right]=\left[\begin{array}{c} A_{HK}{\left({\hat{c}}_{0}^{K-1}\right)}^{T}⊕{\left({\bar{\gamma }}_{0}^{H-1}\right)}^{T}\\ {\left({\hat{c}}_{0}^{K-1}\right)}^{T} \end{array}\right], \end{array}$$

(20) $$\begin{array}{c} {\left(c_{0}^{N-1}\right)}^{T}={\hat{B}}_{N}{\left({\hat{c}}_{0}^{N-1}\right)}^{T}, \end{array}$$

where ${\hat{c}}_{i}\in \{0,1\}$ and ${\hat{B}}_{N}$ is an element-permutation matrix, which is determined by the matrix transformation of (18).

**Proof**

The proof of Corollary 10 is based on Theorem 9 and the linear equation solving theory, which are ignored here.

It is noticed from (18) and (19) that solutions of the error-checking equations tightly depend on the two vectors: ${\bar{\gamma }}_{0}^{H-1}$ and ${\hat{c}}_{0}^{K-1}$. Where ${\bar{\gamma }}_{0}^{H-1}$ is determined by the transformation matrix **P**_*NM*_ and the error-checking vector *γ*_0_^*M*−1^, and ${\hat{c}}_{0}^{K-1}$ is a random vector. In general, based on ${\hat{c}}_{0}^{K-1}$, the number of solutions for the error-checking equations may be up to 2^*K*^, which is a terrible number for decoding. Although the solutions number can be reduced through the checking of (11), it still needs to reduce the solutions number in order to increase efficiency of error checking. To achieve the goal, we further introduce a theorem.

**Theorem 11 .**

For a polar code with code length *N* = 2^*n*^ and frozen node set *V*_*F*_, there exists a positive real number *δ* such that ∀*v*(*i*, *j*) ∈ *V*_*F*_; if (*p*_*v*(*i*,*j*)_(0)/*p*_*v*(*i*,*j*)_(1)) ≥ *δ*, there is

(21) $$\begin{array}{c} \forall v\left(n,j_{k}\right)\in V_{v\left(i,j\right)}^{L}⟹c_{j_{k}}=0, \end{array}$$

where *V*_*v*(*i*,*j*)_^*L*^ is the leaf nodes set of *v*(*i*, *j*), 0 ≤ *j*_*k*_ ≤ *N* − 1, 0 ≤ *k* ≤ |*V*_*v*(*i*,*j*)_^*L*^| − 1, and the value of *δ* is related to the transition probability of the channel and the signal power.

**Proof**

For the proof of Theorem 11 see Appendix D for detail.

Theorem 11 has shown that, with the probability messages of the frozen node and *δ*, we can determine the values of some elements in ${\hat{c}}_{0}^{K-1}$ quickly, by which the freedom degree of ${\hat{c}}_{0}^{K-1}$ will be further reduced. Correspondingly, the number of solutions for the error-checking equations will be also reduced.

Based on above results, we take (14) as an example to show the detailed process of solving the error-checking equations. Through the linear transformation of (18), we have ${\bar{\gamma }}_{0}^{3}=(1,1,1,0)$,

(22) $$\begin{array}{c} A_{4}=\left[\begin{array}{cccc} 1 & 1 & 1 & 0\\ 1 & 1 & 0 & 1\\ 1 & 0 & 1 & 1\\ 0 & 1 & 1 & 1 \end{array}\right], \end{array}$$

(23) $$\begin{array}{c} {\hat{B}}_{8}=\left[\begin{array}{cccccccc} 0 & 0 & 0 & 1 & 0 & 0 & 0 & 0\\ 0 & 0 & 1 & 0 & 0 & 0 & 0 & 0\\ 0 & 1 & 0 & 0 & 0 & 0 & 0 & 0\\ 0 & 0 & 0 & 0 & 0 & 0 & 0 & 1\\ 1 & 0 & 0 & 0 & 0 & 0 & 0 & 0\\ 0 & 0 & 0 & 0 & 0 & 0 & 1 & 0\\ 0 & 0 & 0 & 0 & 0 & 1 & 0 & 0\\ 0 & 0 & 0 & 0 & 1 & 0 & 0 & 0 \end{array}\right]. \end{array}$$

By the element permutation of ${\hat{B}}_{8}$, we further have ${\hat{c}}_{0}^{3}=\left(c_{3},c_{5},c_{6},c_{7}\right)$ and ${\hat{c}}_{4}^{7}=\left(c_{0},c_{1},c_{2},c_{4}\right)$. If (*p*_*v*(0,1)_(0)/*p*_*v*(0,1)_(1)) ≥ *δ*, with the checking of (21), there is (*c*_3_, *c*_5_, *c*_6_, *c*_7_) = (*c*_3_, 0,0, 0), and (*c*_0_, *c*_1_, *c*_2_, *c*_4_) = (*c*_3_ ⊕ 1, *c*_3_ ⊕ 1, *c*_3_ ⊕ 1,0), which imply that the solutions number will be 2. Furthermore, with the checking of (11), we obtain the exact solution *c*_0_^7^ = (0,0, 0,1, 0,0, 0,0); that is, the 4th input node is error.

It is noticed clearly from the above example that, with the checking of (11) and (21), the number of the solutions can be greatly reduced, which make the error checking more efficient. And, of course, the final number of the solutions will depend on the probability messages of the frozen nodes and *δ*.

As the summarization of this section, we given the complete process framework of error checking by solutions of the error-checking equations, which is shown in Algorithm 1.

## 4. Proposed Decoding Algorithm

In this section, we will introduce the proposed decoding algorithm in detail.

### 4.1. Probability Messages Calculating

Probability messages calculating is an important aspect of a decoding algorithm. Our proposed algorithm is different from the SC and BP algorithms, because the probability messages are calculated based on the decoding tree representation of the nodes in the decoder, and for an intermediate node *v*(*i*, *j*) with only one son node *v*(*i* + 1, *j*_*o*_), 0 ≤ *j*_*o*_ ≤ *N* − 1, there is

(24) pv(i,j)(0)=pv(i+1,jo)(0),pv(i,j)(1)=pv(i+1,jo)(1).

While if *v*(*i*, *j*) has two son nodes *v*(*i* + 1, *j*_*l*_) and *v*(*i* + 1, *j*_*r*_), 0 ≤ *j*_*l*_, *j*_*r*_ ≤ *N* − 1, we will have

(25) pv(i,j)(0)=pv(i+1,jl)(0)pv(i+1,jr)(0)     +pv(i+1,jl)(1)pv(i+1,jr)(1),pv(i,j)(1)=pv(i+1,jl)(0)pv(i+1,jr)(1)     +pv(i+1,jl)(1)pv(i+1,jr)(0).

Based on (24) and (25), the probability messages of all the variable nodes can be calculated in parallel, which will be beneficial to the decoding throughput.

### 4.2. Error Correcting

Algorithm 1 in Section 3.3 has provided an effective method to detect errors in the input nodes of the decoder, and, now, we will consider how to correct these errors. To achieve the goal, we propose a method based on modifying the probability messages of the error nodes with constant values according to the maximization principle. Based on the method, the new probability messages of a error node will be given by

(26) qi′(0)=λ0,  qi(0)>qi(1)qi′(0)=1−λ0, otherwise,

and *q*_*i*_′(1) = 1 − *q*_*i*_′(0), where *q*_*i*_′(0), *q*_*i*_′(1) are the new probability messages of the error node *v*(*n*, *i*), and *λ*_0_ is a small nonnegative constant; that is, 0 ≤ *λ*_0_ ≪ 1. Furthermore, we will get the new probability vector of the input nodes as

(27) q0N−1(0)′=(q0(0)′,q1(0)′,…,qN−1(0)′)q0N−1(1)′=(q0(1)′,q1(1)′,…,qN−1(1)′),

where *q*_*i*_(0)′ = *q*_*i*_′(0) and *q*_*i*_(1)′ = *q*_*i*_′(1), if the input node *v*(*n*, *i*) is error; otherwise, *q*_*i*_(0)′ = *q*_*i*_(0) and *q*_*i*_(1)′ = *q*_*i*_(1). Then, probability messages of all the nodes in the decoder will be recalculated.

In fact, when there is only one error indicator vector output from Algorithm 1, that is, |*C*| = 1, after the error correcting and the probability messages recalculation, the estimated source binary vector ${\hat{u}}_{0}^{N-1}$ can output directly by the hard decision of the output nodes. While if |*C*| > 1, in order to minimize the decoding error probability, it needs further research about how to get the optimal error indicator vector.

### 4.3. Reliability Degree

To find the optimal error indicator vector, we will introduce a parameter called* reliability degree* for each node in the decoder. And for a node *v*(*i*, *j*), the reliability degree *ζ* ^*v*(*i*,*j*)^ is given by

(28) $$\begin{array}{c} \zeta^{v(i,j)}=\{\begin{array}{cc} \frac{p_{v(i,j)}\left(0\right)}{p_{v(i,j)}\left(1\right)}, & p_{v(i,j)}\left(0\right)>p_{v(i,j)}\left(1\right)\\ \frac{p_{v(i,j)}\left(1\right)}{p_{v(i,j)}\left(0\right)}, & \text{otherwise}. \end{array} \end{array}$$

The reliability degree indicates the reliability of the node's decision value, and the larger the reliability degree, the higher the reliability of that value. For example, if the probability messages of the node *v*(0,0) in Figure 2 are *p*_*v*(0,0)_(0) = 0.95 and *p*_*v*(0,0)_(1) = 0.05, there is *ζ* ^*v*(*i*,*j*)^ = 0.95/0.05 = 19; that is, the reliability degree of *v*(0,0) = 0 is 19. And in fact, the reliability degree is an important reference parameter for the choice of the optimal error indicator vector, which will be introduced in the following subsection.

### 4.4. Optimal Error Indicator Vector

As aforementioned, due to the existence of |*C*| > 1, correspondingly, one node in the decoder may have multiple reliability degrees. We denote the *k*th reliability degree of node *v*(*i*, *j*) as *ζ*_*k*_^*v*(*i*,*j*)^, value of which depends on the *k*th element of *C*, that is, ${\vec{c}}_{k}$. Based on the definition of reliability degree, we introduce three methods to get the optimal error indicator vector.

The first method is based on the fact that, when there is no noise in the channel, the reliability degree of the node will approach to infinity; that is, *ζ* ^*v*(*i*,*j*)^ → *∞*. Hence, the main consideration is to maximize the reliability degree of all the nodes in decoder, and the target function can be written as

(29) $$\begin{array}{c} \hat{k}=\underset{{\vec{c}}_{k}\in C}{\text{argmax}}\left\{\overset{\text{lo}{\text{g}}_{2}N}{\underset{i=0}{\sum }}\overset{N}{\underset{j=0}{\sum }}\zeta_{k}^{v(i,j)}\right\}, \end{array}$$

where ${\vec{c}}_{\hat{k}}$ is the optimal error indicator vector.

To reduce the complexity, we have further introduced two simplified versions of the above method. On one hand, we just maximize the reliability degree of all the frozen nodes; hence, the target function can be written as

(30) $$\begin{array}{c} \hat{k}=\underset{{\vec{c}}_{k}\in C}{\text{argmax}}\left\{\underset{v(i,j)\in V_{F}}{\sum }\zeta_{k}^{v(i,j)}\right\}. \end{array}$$

On the other hand, we take the maximization of the output nodes' reliability degree as the optimization target, function of which will be given by

(31) $$\begin{array}{c} \hat{k}=\underset{{\vec{c}}_{k}\in C}{\text{argmax}}\left\{\overset{N-1}{\underset{j=0}{\sum }}\zeta_{k}^{v(0,j)}\right\}. \end{array}$$

Hence, the problem of getting the optimal error indicator vector can be formulated as an optimization problem with the above three target functions. What is more is that, with the CRC aided, the accuracy of the optimal error indicator vector can be enhanced. Based on these observations, the finding of the optimal error indicator vector will be divided into the following steps.

1. Initialization: we first get number *L* candidates of the optimal error indicator vector, ${\vec{c}}_{{\hat{k}}_{0}},{\vec{c}}_{{\hat{k}}_{1}},\ldots ,{\vec{c}}_{{\hat{k}}_{L-1}}$, by the formulas of (29) or (30) or (31).
2. CRC-checking: in order to get the optimal error indicator vector correctly, we further exclude some candidates from ${\vec{c}}_{{\hat{k}}_{0}},{\vec{c}}_{{\hat{k}}_{1}},\ldots ,{\vec{c}}_{{\hat{k}}_{L-1}}$ by the CRC-checking. If there is only one valid candidate after the CRC-checking, the optimal error indicator vector will be output directly; otherwise, the remaining candidates will further be processed in step 3.
3. Determination: if there are multiple candidates with a correct CRC-checking, we will further choose the optimal error indicator vector from the remaining candidates of step 2 with the formulas of (29) or (30) or (31).

So far, we have introduced the main steps of proposed decoding algorithm in detail, and, as the summarization of these results, we now provide the whole decoding procedure with the form of pseudocode, as shown in Algorithm 2.

### 4.5. Complexity Analysis

In this section, the complexity of the proposed decoding algorithm is considered. We first investigate the space and time complexity of each step in Algorithm 2, as shown in Table 1.

In Table 1, *O*(*X*_0_), *O*(*X*_1_) are the space and time complexity of Algorithm 1, respectively, and *T*_0_ is the element number of error indicator vectors output by Algorithm 1; that is, *T*_0_ = |*C*|. It is noticed that the complexity of the Algorithm 1 has a great influence on the complexity of the proposed decoding algorithm; hence, we further analyze the complexity of each step of Algorithm 1, and the results are shown in Table 2.

In Table 2, *M* is the number of the frozen nodes, and *T*_1_ is the valid solution number of the error-checking equations after the checking of (21). Hence, we get the space and time complexity of Algorithm 1 as *O*(*X*_0_) = *O*(1) and *O*(*X*_1_) = 2*O*(*M*) + *O*(*MN*) + *O*(*T*_1_(*M* − *K*)*K*) + *O*(*T*_1_ *M*). Furthermore, we can get the space and time complexity of the proposed decoding algorithm as *O*((*T*_0_ + 1)*N*log⁡_2_ *N*) and *O*(2*N*) + *O*((2*T*_0_ + 1)*N*log⁡_2_ *N*) + *O*((*T*_1_ + *N* + 2)*M*) + *O*(*T*_1_ *K*(*N* − *K*)). From these results, we can find that the complexity of the proposed decoding algorithm mainly depends on *T*_0_ and *T*_1_, values of which depend on the different channel condition, as illustrated in our simulation work.

## 5. Simulation Results

In this section, Monte Carlo simulation is provided to show the performance and complexity of the proposed decoding algorithm. In the simulation, the BPSK modulation and the additive white Gaussian noise (AWGN) channel are assumed. The code length is *N* = 2^3^ = 8, code rate *R* is 0.5, and the index of the information bits is the same as [1].

### 5.1. Performance

**Figure 4:**
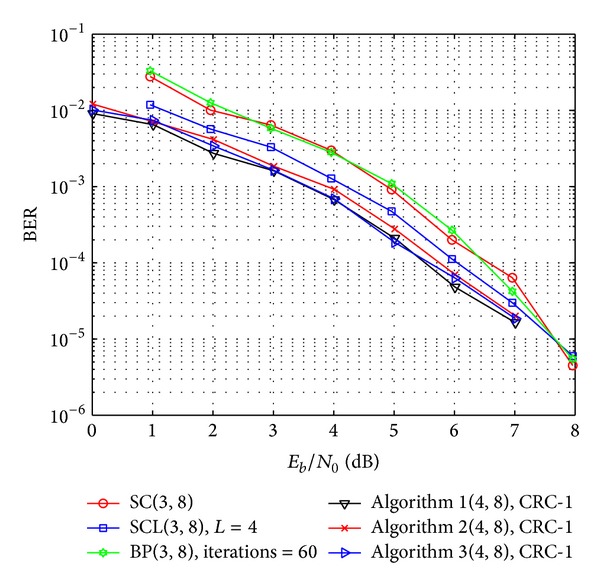
Performance comparison of SC, SCL(*L* = 4), BP (iteration number is 60), and the proposed decoding algorithm. Algorithm 1 means that target function to get the optimal error indicator vector is (29), Algorithm 2 means that the target function is (30), and Algorithm 3 means that the target function is (31). *δ* in Theorem 11 takes the value of 4.

To compare the performance of SC, SCL, BP, and the proposed decoding algorithms, three optimization targets with 1 bit CRC are used to get the optimal error indicator vector in our simulation, and the results are shown in Figure 4.

As it is shown from Algorithms 1, 2, and 3 in Figure 4, the proposed decoding algorithm almost yields the same performance with the three different optimization targets. Furthermore, we can find that, compared with the SC, SCL, and BP decoding algorithms, the proposed decoding algorithm achieves better performance. Particularly, in the low region of signal to noise ratio (SNR), that is, *E*_*b*_/*N*_0_, the proposed algorithm provides a higher SNR advantage; for example, when the bit error rate (BER) is 10^−3^, Algorithm 1 provides SNR advantages of 1.3 dB, 0.6 dB, and 1.4 dB, and when the BER is 10^−4^, the SNR advantages are 1.1 dB, 0.5 dB, and 1.0 dB, respectively. Hence, we can conclude that performance of short polar codes could be improved with the proposed decoding algorithm.

**Figure 5:**
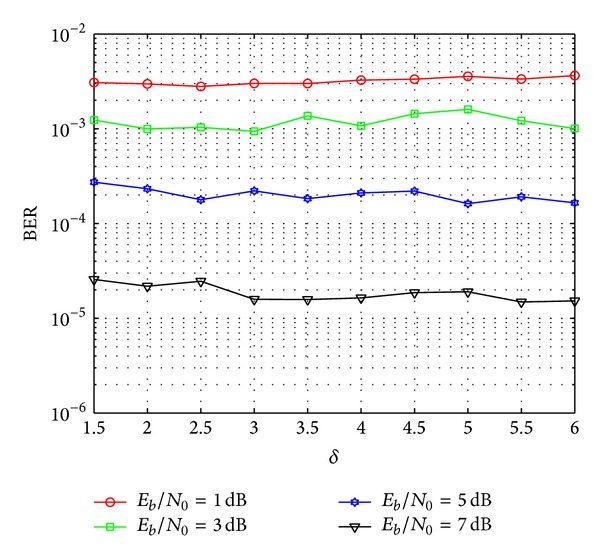
Performance of proposed decoding algorithm with different *δ*.

In addition, it is noted from Theorem 11 that the value of *δ* depended on the transition probability of the channel and the signal power will affect the performance of the proposed decoding algorithm. Hence, based on Algorithm 1 in Figure 4, the performance of our proposed decoding algorithm with different *δ* and SNR is also simulated, and the results are shown in Figure 5. It is noticed that the optimal values of *δ* according to *E*_*b*_/*N*_0_ = 1 dB, *E*_*b*_/*N*_0_ = 3 dB, *E*_*b*_/*N*_0_ = 5 dB, and *E*_*b*_/*N*_0_ = 7 dB are 2.5, 3.0, 5.0, and 5.5, respectively.

### 5.2. Complexity

**Figure 6:**
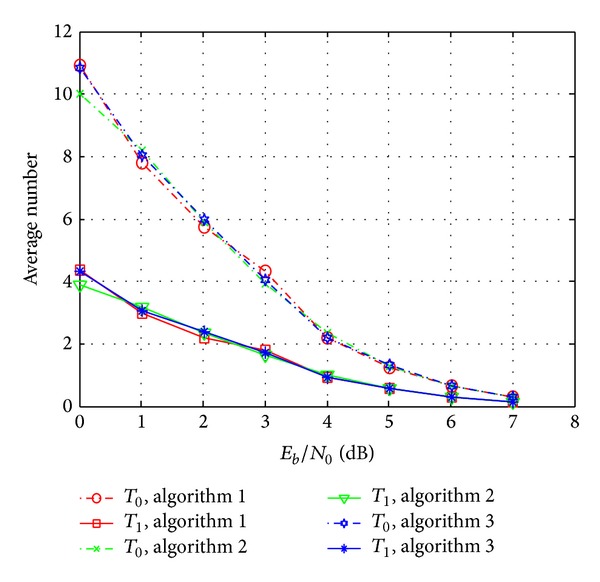
Average number of parameters *T*_0_ and *T*_1_ with *δ* = 4.

To estimate the complexity of the proposed decoding algorithm, the average numbers of parameters *T*_0_ and *T*_1_ indicated in Section 4.5 are counted and shown in Figure 6.

**Figure 7:**
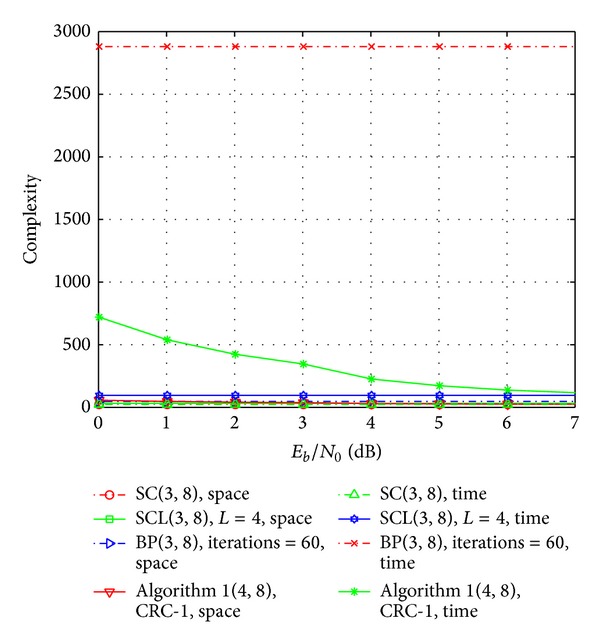
Space and time complexity comparison of SC, SCL(*L* = 4), BP (iteration number is 60), and Algorithm 1 (*δ* = 4).

It is noticed from Figure 6 that, with the increasing of the SNR, the average numbers of parameters *T*_0_ and *T*_1_ are sharply decreasing. In particular, we can find that, in the high SNR region, both of the *T*_0_ and *T*_1_ are approaching to a number less than 1. In this case, the space complexity of the algorithm will be *O*(*N*log⁡_2_ *N*), and the time complexity approaches to *O*(*NM*). In addition, we further compare the space and time complexity of Algorithm 1 (*δ* = 4) and SC, SCL (*L* = 4), and BP decoding algorithm, results of which are shown in Figure 7. It is noticed that, in the high SNR region, the space complexity of the proposed algorithm is almost the same as that of SC, SCL, and BP decoding algorithm, and the space complexity of the proposed algorithm will be close to *O*(*NM*). All of the above results have suggested the effectiveness of our proposed decoding algorithm.

## 6. Conclusion

In this paper, we proposed a parallel decoding algorithm based on error checking and correcting to improve the performance of the short polar codes. To enhance the error-correcting capacity of the decoding algorithm, we derived the error-checking equations generated on the basis of the frozen nodes, and through delving the problem of solving these equations, we introduced the method to check the errors in the input nodes by the solutions of the equations. To further correct those checked errors, we adopted the method of modifying the probability messages of the error nodes with constant values according to the maximization principle. Due to the existence of multiple solutions of the error-checking equations, we formulated a CRC-aided optimization problem of finding the optimal solution with three different target functions, so as to improve the accuracy of error checking. Besides, in order to increase the throughput of decoding, we used a parallel method based on the decoding tree to calculate probability messages of all the nodes in the decoder. Numerical results showed that the proposed decoding algorithm achieves better performance than that of the existing decoding algorithms, where the space and time complexity were approaching to *O*(*N*log_2_ *N*) and *O*(*NM*) (*M* is the number of frozen nodes) in the high signal to noise ratio (SNR) region, which suggested the effectiveness of the proposed decoding algorithm.

It is worth mentioning that we only investigated the error correcting for short polar codes, while for the long-length codes, the method in this paper will yield higher complexity. Hence, in future, we will extend the idea of error correcting in this paper to the research of long code length in order to further improve the performance of polar codes.

## Figures and Tables

**Algorithm 1:**
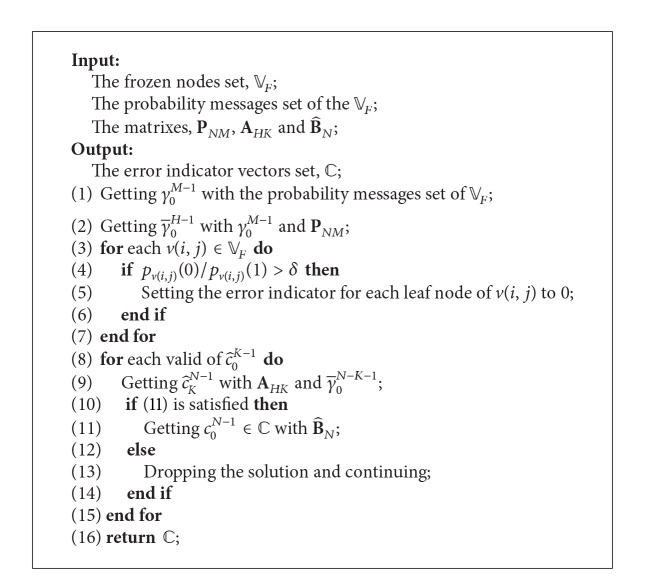
Error checking for decoding.

**Algorithm 2:**
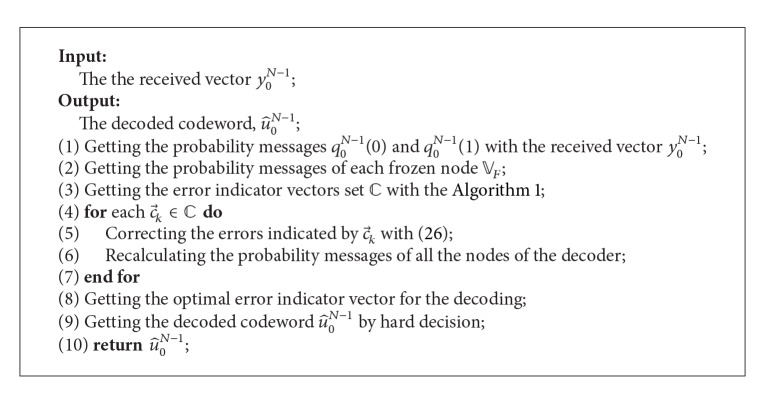
Decoding algorithm based on error checking and correcting.

**Table 1:** The space and time complexity of each step in Algorithm 2.

Step number in Algorithm 2	Space complexity	Time complexity
(1)	*O*(1)	*O*(*N*)
(2)	*O*(*N*log_2_⁡*N*)	*O*(*N*log_2_⁡*N*)
(3)	*O*(*X*_0_)	*O*(*X*_1_)
(4)–(7)	*O*(*T*_0_ *N*log_2_⁡*N*)	*O*(*T*_0_ *N*log_2_⁡*N*)
(8)	*O*(1)	*O*(*T*_0_ *N*log_2_⁡*N*) or *O*(*T*_0_ *N*)
(9)	*O*(1)	*O*(*N*)

**Table 2:** The space and time complexity of each step in Algorithm 1.

Step number in Algorithm 1	Space complexity	Time complexity
(1)	*O*(1)	*O*(*M*)
(2)	*O*(1)	*O*(*M*)
(3)–(7)	*O*(1)	*O*(*MN*)
(8)–(15)	*O*(1)	*O*(*T*_1_(*M* − *K*)*K*) + *O*(*T*_1_ *M*)

## Appendix

### A. Proof of Theorem 1

We can get the inverse of **F**_2_ through the linear transformation of matrix; that is $F_{2}^{-1}=\left[\begin{array}{cc} 1 & 0\\ 1 & 1 \end{array}\right]$. Furthermore, we have

(A.1) (F2⊗2)−1=[F202F2F2]−1=[F2−102−F2−1F2F2−1F2−1]=[F202F2F2]=F2⊗2.

Based on the mathematical induction, we will have

(A.2) $$\begin{array}{c} {\left(F_{2}^{⊗n}\right)}^{-1}=F_{2}^{⊗n}. \end{array}$$

The inverse of **G**_*N*_ can be expressed as

(A.3) $$\begin{array}{c} G_{N}^{-1}={\left(B_{N}F_{2}^{⊗n}\right)}^{-1}={\left(F_{2}^{⊗n}\right)}^{-1}B_{N}^{-1}=F_{2}^{⊗n}B_{N}^{-1}. \end{array}$$

Since **B**_*N*_ is a bit-reversal permutation matrix, by elementary transformation of matrix, there is **B**_*N*_^−1^ = **B**_*N*_. Hence, we have

(A.4) $$\begin{array}{c} G_{N}^{-1}=F_{2}^{⊗n}B_{N}. \end{array}$$

It is noticed from* Proposition* 16 of [1] that **F**_2_^⊗*n*^ **B**_*N*_ = **B**_*N*_ **F**_2_^⊗*n*^; therefore there is

(A.5) $$\begin{array}{c} G_{N}^{-1}=G_{N}. \end{array}$$

### B. Proof of Theorem 4

We assume the leaf nodes number of the frozen node *v*(*i*, *j*) is *Q*; that is, *Q* = |*V*_*v*(*i*,*j*)_^*L*^|. If *Q* = 2, based on ((25)), there is

(B.1) pv(i,j)(0)=pv0(0)pv1(0)+pv0(1)pv1(1)pv(i,j)(1)=pv0(0)pv1l(1)+pv0(1)pv1(0),

where *v*_0_, *v*_1_ ∈ *V*_*v*(*i*,*j*)_^*L*^. Based on the above equations, we have

(B.2) pv(i,j)(0)−pv(i,j)(1) =(pv0(0)−pv0(1))(pv1(0)−pv1(1)).

Therefore, by the mathematical inducing, when *Q* > 2, we will have

(B.3) $$\begin{array}{c} p_{v(i,j)}\left(0\right)-p_{v(i,j)}\left(1\right)=\overset{Q-1}{\underset{k=0}{\prod }}\left(p_{v_{k}}\left(0\right)-p_{v_{k}}\left(1\right)\right), \end{array}$$

where *v*_*k*_ ∈ *V*_*v*(*i*,*j*)_^*L*^.

To prove Theorem 4, we assume that the values of all the nodes in *V*_*v*(*i*,*j*)_^*L*^ are set to 0 without generality. That is to say, when the node *v*_*k*_ ∈ *V*_*v*(*i*,*j*)_^*L*^ is right, there is *p*_*v*_*k*__(0) > *p*_*v*_*k*__(1). Hence, based on the above equation, when the probability messages of *v*(*i*, *j*) do not satisfy the reliability condition, that is, *p*_*v*(*i*,*j*)_(0) − *p*_*v*(*i*,*j*)_(1) ≤ 0, there must exist a subset *V*_*v*(*i*,*j*)_^*LO*^⊆*V*_*v*(*i*,*j*)_^*L*^, and |*V*_*v*(*i*,*j*)_^*LO*^| is an odd number, such that

(B.4) $$\begin{array}{c} \forall v_{k}\in V_{v\left(i,j\right)}^{LO}⟶p_{v_{k}}\left(0\right)\leq p_{v_{k}}\left(1\right). \end{array}$$

While if *p*_*v*(*i*,*j*)_(0) − *p*_*v*(*i*,*j*)_(1) > 0, there must exist a subset *V*_*v*(*i*,*j*)_^*LE*^⊆*V*_*v*(*i*,*j*)_^*L*^, and |*V*_*v*(*i*,*j*)_^*LE*^| is an even number, such that

(B.5) $$\begin{array}{c} \forall v_{k}\in V_{v\left(i,j\right)}^{LE}⟶p_{v_{k}}\left(0\right)\leq p_{v_{k}}\left(1\right). \end{array}$$

So the proof of Theorem 4 is completed.

### C. Proof of Theorem 9

It is noticed from ((1)) that the coefficient vector of error-checking equation generated by the frozen nodes in the leftmost column is equal to one column vector of **G**_*N*_^−1^, denoted as ${\vec{g}}_{i}$, 0 ≤ *i* ≤ *N* − 1. For example, the coefficient vector of error-checking equation generated by *v*(0,0) is equal to ${\vec{g}}_{1}={\left(1,1,1,1,1,1,1,1\right)}^{T}$. Hence, based on the proof of Theorem 1, we have

(C.1) $$\begin{array}{c} \mathrm{rank}\left(E_{MN}\right)\geq N-K,\\ \mathrm{rank}\left(\left[\begin{array}{cc} E_{MN} & {\left(\gamma_{0}^{M-1}\right)}^{T} \end{array}\right]\right)\geq N-K. \end{array}$$

In view of the process of polar encoding, we can find that the frozen nodes in the intermediate column are generated by the linear transformation of the frozen nodes in the leftmost column. That is to say, error-checking equation generated by the frozen nodes in the intermediate column can be linear expressed by the error-checking equation generated by the frozen nodes in the leftmost column. Hence, we further have

(C.2) $$\begin{array}{c} \mathrm{rank}\left(E_{MN}\right)\leq N-K,\\ \mathrm{rank}\left(\left[\begin{array}{cc} E_{MN} & {\left(\gamma_{0}^{M-1}\right)}^{T} \end{array}\right]\right)\leq N-K. \end{array}$$

Therefore, the proof of ((17)) is completed.

### D. Proof of Theorem 11

To prove Theorem 11, we assume that the real values of all the input nodes are 0 without generality. Given the conditions of transition probability of the channel and constraint of the signal power, it can be easily proved that there exists a positive constant *β*_0_ > 1, such that

(D.1) $$\begin{array}{c} \forall v\left(n,k\right)\in V_{I}⟹\frac{1}{\beta_{0}}\leq \frac{p_{v(n,k)}\left(0\right)}{p_{v(n,k)}\left(1\right)}\leq \beta_{0}, \end{array}$$

where *v*(*n*, *k*) is an input node and *V*_*I*_ is the input nodes set of the decoder. That is to say, for a frozen node *v*(*i*, *j*) with a leaf nodes set *V*_*v*(*i*,*j*)_^*L*^, we have

(D.2) $$\begin{array}{c} \forall v_{k}\in V_{v\left(i,j\right)}^{L}⟹\frac{1}{\beta_{0}}\leq \frac{p_{v_{k}}\left(0\right)}{p_{v_{k}}\left(1\right)}\leq \beta_{0}. \end{array}$$

Based on ((25)) and the decoding tree of *v*(*i*, *j*), we have the probability messages of *v*(*i*, *j*) as

(D.3) pv(i,j)(0) =∑m=0Q/2−1∑∀{k0,…,k2m−1}⊆{0,…,Q−1}∏l=02m−1pvkl(1)∏r=0,0≤kr≤Q−1,kr∉{k0,…,k2m−1}Q−2m−1pvkr(0),pv(i,j)(1) =∑m=0Q/2−1∑∀{k0,…,k2m}⊆{0,…,Q−1}∏l=02mpvkl(1)∏r=0,0≤kr≤Q−1,kr∉{k0,…,k2m}Q−2m−1pvkr(0),

where *v*_*k*_*l*__, *v*_*k*_*r*__ ∈ *V*_*v*(*i*,*j*)_^*L*^. Hence, we further have

(D.4) pv(i,j)(0)pv(i,j)(1) =1+∑m=1Q/2−1∑∀{k0,…,k2m−1}⊆{0,…,Q−1}∏l=02m−1(pvkl(0)/pvkl(1))∑m=0Q/2−1∑∀{k0,…,k2m}⊆{0,…,Q−1}∏l=02m(pvkl(0)/pvkl(1)).

With definition of variables *φ*_0_ = *p*_*v*_0__(0)/*p*_*v*_0__(1), *φ*_1_ = *p*_*v*_1__(0)/*p*_*v*_1__(1),…, *φ*_*Q*−1_ = *p*_*v*_*Q*−1__(0)/*p*_*v*_*Q*−1__(1), 1/*β*_0_ ≤ *φ*_0_, *φ*_1_,…, *φ*_*Q*−1_ ≤ *β*_0_, the above equation will be written as

(D.5) pv(i,j)(0)pv(i,j)(1)=f(φ0,φ1,…,φQ−1)=(1+⋯+φQ−2φQ−1+φ0φ1φ2φ3  +⋯+φQ−4φQ−3φQ−2φQ−1+⋯) ×(φ0+⋯+φQ−1+φ0φ1φ2   +⋯+φQ−3φQ−2φQ−1+⋯)−1.

To take the derivative of *f*(*φ*_0_, *φ*_1_,…, *φ*_*Q*−1_), we further define functions as

(D.6) h(φ0,φ1,…,φQ−1) =1+φ0φ1+⋯+φQ−2φQ−1  +φ0φ1φ2φ3+⋯+φQ−4φQ−3φQ−2φQ−1+⋯,g(φ0,φ1,…,φQ−1) =φ0+⋯+φQ−1+φ0φ1φ2+⋯  +φQ−3φQ−2φQ−1+⋯.

Then, the derivative of *f*(*φ*_0_, *φ*_1_,…, *φ*_*Q*−1_) with respect to *φ*_*k*_ will be

(D.7) ∂f∂φk=(∂h/∂φk)g−(∂g/∂φk)hg2=ggφk=0−hhφk=0g2=gφk=02−hφk=02g2,

where *g*_*φ*_*k*_=0_ = *g*(*φ*_0_,…, *φ*_*k*−1_, 0, *φ*_*k*+1_,…, *φ*_*Q*−1_) and *h*_*φ*_*k*_=0_ = *h*(*φ*_0_,…, *φ*_*k*−1_, 0, *φ*_*k*+1_,…, *φ*_*Q*−1_). Based on solution of the equations (∂*f*/∂*φ*_0_) = 0, (∂*f*/∂*φ*_1_) = 0,…, and (∂*f*/∂*φ*_*Q*−1_) = 0, we get the extreme value point of *f*(*φ*_0_, *φ*_1_,…, *φ*_*Q*−1_) as *φ*_0_ = *φ*_1_ = ⋯ = *φ*_*Q*−1_ = 1. Based on the analysis of the monotonicity of *f*(*φ*_0_, *φ*_1_,…, *φ*_*Q*−1_), we can get the maximum value as $\delta =f\left(\underset{Q}{\underset{︸}{\beta_{0},\beta_{0},\ldots ,\beta_{0}}}\right)$. What is more, we can also get that when *f*(*φ*_0_, *φ*_1_,…, *φ*_*Q*−1_) ≥ *δ*, there is *φ*_0_ > 1, *φ*_1_ > 1,…, and *φ*_*Q*−1_ > 1. That is to say, when (*p*_*v*(*i*,*j*)_(0)/*p*_*v*(*i*,*j*)_(1)) ≥ *δ*, we will have *p*_*v*_0__(0) > *p*_*v*_0__(1), *p*_*v*_1__(0) > *p*_*v*_1__(1),…, and *p*_*v*_*Q*−1__(0) > *p*_*v*_*Q*−1__(1); that is, there is no error in *V*_*v*(*i*,*j*)_^*L*^. So the proof of Theorem 11 is completed.
